# Reliability and validity of the Turkish version of the extended Barcelona Music Reward Questionnaire

**DOI:** 10.1371/journal.pone.0347517

**Published:** 2026-06-18

**Authors:** Ozgenur Cetinbag-Kuzu, Zehra Aydoğan, Mustafa Kürşat Gökcan

**Affiliations:** 1 Department of Audiology, Faculty of Health Sciences, Sakarya University, Sakarya, Turkey; 2 Department of Audiology, Faculty of Health Sciences, Ankara University, Ankara, Turkey; 3 Department of Otorhinolaryngology, Faculty of Medicine, Ankara University, Ankara, Turkey; Annamalai University Faculty of Fine Arts, INDIA

## Abstract

Individual differences in musical reward have increasingly been studied in neuroscience and music research. However, there are limited validated tools for evaluating musical reward with multidimensional measures in Turkish-speaking populations. The extended Barcelona Music Reward Questionnaire offers a comprehensive assessment of how individuals experience pleasure from music. This study aims to investigate the validity and reliability of the Turkish version of the extended Barcelona Music Reward Questionnaire (eBMRQ-TR). A total of 266 Turkish-speaking adults aged 18–65 completed the eBMRQ-TR using an online platform. This study included 189 non-musicians, 57 amateur musicians, and 20 professional musicians. Construct validity was investigated using exploratory and confirmatory factor analyses. Internal consistency was assessed using Cronbach’s alpha and McDonald’s omega. Test-retest reliability over a three-week period was evaluated in a subsample of 65 participants through intraclass correlation coefficients (ICCs). Exploratory analyses were performed to define group differences in musicianship, gender, and age. The eBMRQ-TR comprised 24 items across six factors, accounting for 52.41% of the total variance. Confirmatory factor analysis indicated acceptable model fit [χ² (237) = 628, CFI = .946, TLI = .938, RMSEA = .079, SRMR = .057], supporting a six-factor structure for the eBMRQ-TR. The total eBMRQ-TR showed excellent internal consistency (α = .92; ω = .93). The test-retest reliability of the total eBMRQ-TR showed satisfactory temporal consistency, with an ICC of  .80. Musicians demonstrated higher total eBMRQ-TR scores than non-musicians (*p* < .001, *η²* = .11). The eBMRQ-TR is a valid and reliable instrument for measuring musical reward in Turkish-speaking adults. As a multidimensional measure, it could inform future studies of individual differences in musical engagement, particularly for research on musical reward and music perception.

## Introduction

Music can elicit strong emotional and rewarding responses [1–3]. Music is not typically considered a primary biological reward, such as food; however, it robustly activates neural mechanisms involved in reward processing and reinforcement learning [4,5]. The neurological underpinnings of music-induced pleasure involve the engagement of reward, motivation, and emotion-related brain circuits in response to pleasant music [6]. Thus, musical pleasure may influence how individuals experience and respond to music [4].

Neuroimaging and neurochemical research indicate that listening to pleasurable music activates reward-related brain networks, such as the nucleus accumbens (NAc), ventral tegmental area (VTA), and mesocorticolimbic dopaminergic systems [5]. Listening to pleasurable music is also influenced by the functional connections between the auditory cortex and reward-related brain areas, including interactions between sound perception and reward evaluation [6–9]. Auditory-reward circuits are engaged by predictive reactions to musical structure, including rhythmic and harmonic predictions, contributing to the experience of pleasure [3,4,10,11]. Moreover, musical training is associated with enhanced connectivity between the auditory system and reward-related regions [6,12], which may increase musicians’ sensitivity to musical reward [13].

Evidence suggests that the mechanisms underlying musical reward are highly complicated, exhibiting significant inter-individual differences that make it challenging to characterize this concept objectively [4,14]. Individual experiences of musical pleasure vary and are influenced by several social and personal characteristics [15], including empathy [16], musical experience [17], cognitive ability [18], and cultural or situational context [19,20]. Some listeners experience strong physiological or affective responses, whereas others report limited pleasure or even a neutral response [21,22]. Identifying these individual differences may help elucidate how aesthetic experiences, such as listening to music, interact with reward- and emotion-related brain regions [6]. Addressing these individual differences, Mas-Herrero et al. [13] developed the Barcelona Music Reward Questionnaire (BMRQ), a psychometric tool intended to measure multiple aspects of the reward associated with music listening. The BMRQ evaluates musical reward in detail as a multifaceted construct with five domains: Musical Seeking, Emotion Evocation, Mood Regulation, Social Reward, and Sensory-Motor. Musical Seeking reflects the motivation to actively pursue and engage in musical activities, such as attending performances; Emotion Evocation conveys the ability of music to elicit pertinent emotional reactions in individuals; Mood Regulation evaluates the use of music to control mood; Social Reward captures how music fosters and improves social interaction; lastly, spontaneous motor tendencies like synchronization or movement in response to rhythmic inputs are referred to as Sensory-Motor [13].

Following its initial development, the BMRQ and its extended version have been adapted and validated in several linguistic and cultural populations, such as French [23], Chinese [24], Brazilian Portuguese [25], Danish [26], and Italian [27]. Evidence from these studies proves the BMRQ’s psychometric strength and cross-cultural validity, highlighting its suitability for examining the cultural, psychological, and biological factors of musical pleasure. More specifically, Cardona et al. [28] developed the extended Barcelona Music Reward Questionnaire (eBMRQ), which added a sixth factor, i.e., Absorption in Music, to the foundational framework. This sixth factor reflects the ability to become deeply engaged and emotionally immersed in musical experiences, which was based on the Absorption in Music Scale developed by Sandstrom and Russo [29]. The Absorption in Music scale measures the intensity and quality of emotions evoked by music, as well as the degree to which an individual is involved and immersed in the musical experience [29]. Additionally, absorption is associated with listening preferences and musical experiences, influencing the time individuals devote to music and the personal significance they attribute to it [29]. Overall, including this dimension into the BMRQ broadens the explanatory scope of the original model, allowing a more comprehensive investigation of individual differences in musical pleasure and engagement. However, few studies have demonstrated good factorial validity and internal consistency for the eBMRQ across populations [27,28].

Although the eBMRQ has demonstrated good psychometric performance [13, 27, 28], it has not yet been translated or validated into Turkish, highlighting a gap in the assessment of musical reward sensitivity. The Turkish version of the eBMRQ is expected to pioneer the examination of the relationship between musical experiences and reward mechanisms in Türkiye, thereby offering valuable contributions to research on musical reward and potentially relevant to clinical contexts. The objective of this study was to adapt and validate the eBMRQ for Turkish-speaking adults and to examine its factorial structure, reliability, and construct validity.

## Materials and methods

### Study design

This is a cross-sectional, descriptive psychometric validation study. The study protocol was approved by the Ankara University Ethics Committee (Approval No: İ09-845-25; October 24, 2025). All procedures were conducted in accordance with the Declaration of Helsinki, and informed consent was obtained electronically from all participants before data collection. Identifiable information, such as names, dates of birth, and contact details, was collected for administrative reasons and then removed before analysis. All statistical analyses were performed using fully anonymized data, with no identifiable information retained. Participants were recruited between 30 October 2025 and 1 January 2026.

### Participants

A total of 278 participants completed the questionnaire. However, eight participants with self-reported hearing loss and four participants who did not provide their date of birth were excluded. Accordingly, 266 (144 females, 122 males) Turkish-speaking adults aged 18–65 were recruited in the final analyses. Regarding the inclusion criteria, participants were required to be aged 18–65, native Turkish speakers, and without hearing loss. This study included participants with self-reported normal hearing and had no diagnosed hearing loss or neurological conditions. Regarding the exclusion criteria, participants were younger than 18 or older than 65, not proficient in Turkish, or had hearing loss. Participants reported demographic characteristics (age, gender, and date of birth) and self-categorized their musicianship status as non-musician, amateur musician, or professional musician. The eBMRQ-TR was administered via an online platform (Google Forms) to the general population. To evaluate test-retest reliability, a subsample of 65 responders (approximately 25% of the sample) recompleted the eBMRQ-TR after a three-week interval.

### Extended Barcelona Music Reward Questionnaire (eBMRQ)

The extended Barcelona Music Reward Questionnaire, i.e., eBMRQ [28], is a 24-item self-report measure developed to capture musical reward experiences and pleasure related to music in individuals. Specifically, this instrument extended the original Barcelona Music Reward Questionnaire [13] by introducing a sixth dimension, i.e., Absorption in Music, which reflects immersive psychological involvement and attentional engagement during music listening. The eBMRQ consisted of six subscales representing aspects of musical reward: Musical Seeking (MS), Emotion Evocation (EE), Mood Regulation (MR), Social Reward (SR), Sensory-Motor (SM), and Absorption in Music (AM). Collectively, these dimensions represented motivational, emotional, social, sensorimotor, and immersive facets of engagement with music. Responses were collected on a five-point Likert scale ranging from *strongly disagree* (1) to *strongly agree* (5), with higher scores indicating stronger sensitivity to musical reward. Total and subscale scores were calculated as the average of related item responses, and the total score indicated overall sensitivity to musical reward.

### Translation and adaptation procedure

Permission to use the original scale in the Turkish adaptation and validation study was obtained from Prof. Antoni Rodríguez-Fornells, one of the developers of the original instrument. The procedure comprised forward-backward translation [30,31], expert review, and pilot testing to achieve conceptual and cultural equivalence with the original instrument. In the initial stage, two bilingual translators (native Turkish speakers with professional proficiency in English) translated the eBMRQ from English into Turkish. The two forward translations were then compared and synthesized into a single primary Turkish version through consensus, with attention to linguistic and cultural relevance. Subsequently, the primary Turkish version was back-translated into English by a bilingual expert proficient in both languages and unfamiliar with the original instrument. This back-translated version was compared with the original English eBMRQ to identify any discrepancies, and minor revisions were made to enhance semantic alignment and conceptual consistency. All items were thoroughly reviewed and, if needed, revised to confirm conceptual clarity and linguistic suitability prior to pilot testing and subsequent main assessment of validity and reliability. Additionally, items 2 and 5 were carefully examined during the translation and cultural adaptation process and revised as needed to ensure clarity of concepts and proper language use before being incorporated into the final version of the instrument. Prior to data collection, the primary Turkish version was pilot-tested with 10 adults to assess clarity and feasibility. Feedback from the pilot test supported the appropriateness of the items, and only a few wording refinements were made before finalizing the instrument. The finalized Turkish version of the eBMRQ was subsequently established in the main study (see S1 File for the Turkish version of the eBMRQ).

### Statistical analysis

All statistical analyses were done using IBM SPSS Statistics (version 31.0) and Jamovi 2.6.45. Categorical variables were presented using frequencies and percentages. Continuous variables with a normal distribution were described as means ± standard deviations (*M* ± *SD*). No missing data were observed across the variables included in the analysis. Data screening revealed no extreme outliers, and all participants were included in the analyses. Items 2 and 5 were reverse-coded before calculating subscale and total scores due to their negative meaning, in accordance with the original BMRQ developmental study by Mas-Herrero et al. (2013). The normality of the data was examined using the Shapiro–Wilk test, with evaluation of skewness and kurtosis. Sample size calculation was performed using standard criteria based on the ratio of participants to measured variables [32]. Consistent with this, 10 individuals per indicator variable was an accepted ratio [33, 34]. Accordingly, the current sample size exceeded the recommended criteria for factor analysis, yielding an approximate ratio of 11 participants per item (266 participants in total).

Item-level analyses involved evaluating corrected item-total correlations and assessing potential floor and ceiling effects. Corrected item-total correlations are commonly regarded as adequate item performance if they are  .30 or higher [35]. The internal consistency of the total eBMRQ-TR and its subscales was assessed employing both Cronbach’s alpha (α) and McDonald’s omega (ω) coefficients [36,37]. Cronbach’s alpha provides a classical estimate of internal consistency based on inter-item correlations [36], while McDonald’s omega offers a model-based reliability estimate that accounts for factor loadings and error variances [37,38]. Both coefficients were evaluated based on widely accepted thresholds: ≥ .60 was considered acceptable, consistent with psychometric standards for multidimensional scales [35,37,39].

To identify the factor structure of the eBMRQ-TR, an Exploratory Factor Analysis (EFA) was conducted applying the Principal Axis Factoring extraction method with Oblimin rotation. The Kaiser-Meyer-Olkin (KMO) measure and Bartlett’s test of sphericity were used to assess sampling adequacy and factorability. Eigenvalues, visual examination of the scree plot, and theoretical considerations reported factor retention decisions. Subsequently, factor structure was examined using Confirmatory Factor Analysis (CFA) in Jamovi. Model fit was investigated via multiple fit indices, including the chi-square statistic (χ²/df), Comparative Fit Index (CFI), Tucker–Lewis Index (TLI), Normed Fit Index (NFI), Root Mean Square Error of Approximation (RMSEA), and Standardized Root Mean Square Residual (SRMR). According to recommended model fit indices, χ²/df ≤ 3 indicates acceptable fit [40]. RMSEA and SRMR values ≤ .08 demonstrate acceptable fit; CFI, TLI, and NFI values ≥ .95 represent good model fit [41]. Standardized factor loadings (λ) were reported to assess the interpretation of the factor structure. According to established guidelines, loadings below  .40 are regarded as weak,  .40 –  .59 moderate, and ≥  .60 strong [32,42]. Moreover, convergent and discriminant validity were assessed using Composite Reliability (CR) and Average Variance Extracted (AVE) from the CFA results, and discriminant validity was also analyzed through the Fornell-Larcker criterion. Test-retest reliability was evaluated in a subsample of 65 participants who recompleted the eBMRQ-TR after three weeks, with the Intraclass Correlation Coefficient (ICC). A two-way fixed-effects model using a consistency definition and single measures, ICC (3,1), was employed, as the same questionnaire was given to the same participants at both time points. This model evaluates the consistency of individual scores across multiple assessments. According to established guidelines, ICC values were interpreted as poor (<  .50), moderate (.50 –  .75), good (.75 –  .90), or excellent (>  .90) for reliability [43].

Exploratory group comparisons were performed to identify variations in eBMRQ-TR scores across demographic characteristics (age and gender) and musicianship status. Gender-related comparisons were performed using independent-samples *t* tests, while one-way analyses of variance (ANOVA) were used to explore differences across age and musicianship when assumptions of normality were met. Post hoc analyses were used to examine pairwise comparisons when statistically significant group differences were found. Tukey’s Honestly Significant Difference (HSD) test was used for parametric tests. Effect sizes were calculated using Cohen’s d and η²/partial η², as appropriate [44]. Construct validity was evaluated via correlation analyses with related constructs, using Spearman’s rank correlation coefficient (*ρ*) to assess the association between age and total eBMRQ scores. Statistical significance was set at *p* < .05 for all analyses, and all tests were two-tailed.

## Results

The sample included 266 Turkish-speaking adults, aged from 18 to 65 (*M ± SD* = 38.3 ± 12.1 years). Participants showed a relatively balanced gender distribution (*n* = 144 females, *n* = 122 males) and age distribution (*n* = 132 aged ≤ 35 years; *n* = 134 aged > 35 years). Participants also varied in musicianship status (non-musician, amateur musician, and professional musician). Demographic characteristics of the individuals, including age, gender, and musicianship status, are represented in Table 1.

**Table 1:** Demographic characteristics of the participants.

Variable	*n* (%) / *M ± SD*
Age (*M ± SD;* years)	38.3 ± 12.1 (18–65)
Gender *n* (%) Female Male	144 (54.3%) 122 (45.7%)
Musicianship status *n* (%) Non-musician Amateur musician Professional musician	189 (71.1%) 57 (21.4%) 20 (7.5%)

Note: *n* (%) = frequency (percentage).

### Item analyses

Item-level analyses were performed to assess the psychometric quality of the eBMRQ-TR items and their subscales. Corrected item-total correlations ranged from  .29 to  .73. Only one item demonstrated a slightly lower correlation (*r* = .29), while all remaining items indicated acceptable to strong associations with the total eBMRQ-TR score (*r* ≥ .35). Across all items, no substantial floor effects were found, with minimum response rates below 15%, in agreement with recommended standards [45]. Ceiling effects were noted for certain positively worded items; however, response patterns were adequately distributed over the Likert scale. The total eBMRQ-TR score was 3.70 ± .7 (*M ± SD*). Item-level descriptive statistics, corrected item-total correlations, and subscale characteristics, including means and standard deviations, are reported in Table 2.

**Table 2:** Item descriptive statistics and item-total correlations for the eBMRQ-TR.

Item	Subscale	*M*	*SD*	Corrected item-total correlation (*r)*
Item 1	SR	3.88	1.08	.41
Item 2	MS	3.83	1.36	.39
Item 3	EE	4.21	.98	.35
Item 4	MR	4.18	1.04	.53
Item 5	SM	3.72	1.38	.29
Item 6	AM	4.05	1.14	.72
Item 7	SR	3.62	1.22	.64
Item 8	MS	3.65	1.22	.52
Item 9	EE	4.47	.80	.53
Item 10	MR	4.30	.89	.62
Item 11	SM	3.03	1.28	.51
Item 12	AM	2.99	1.26	.63
Item 13	MS	3.24	1.23	.43
Item 14	EE	3.65	1.32	.49
Item 15	SR	3.42	1.36	.59
Item 16	MR	4.00	1.05	.73
Item 17	SM	4.22	1.01	.67
Item 18	AM	3.32	1.31	.65
Item 19	SR	3.59	1.27	.64
Item 20	MS	2.18	1.24	.48
Item 21	EE	3.38	1.27	.61
Item 22	MR	4.23	.93	.69
Item 23	SM	4.00	1.07	.71
Item 24	AM	3.71	1.18	.70

Note: Item scores are reported as mean ± standard deviations. MS = Musical Seeking; EE = Emotion Evocation; MR = Mood Regulation; SR = Social Reward; SM = Sensory–Motor; AM = Absorption in Music.

### Exploratory Factor Analyses (EFA) and Confirmatory Factor Analyses (CFA)

Prior to exploratory factor analysis (EFA), sampling adequacy and factorability were evaluated. The Kaiser–Meyer–Olkin (KMO) measure showed high sampling adequacy (KMO = .92), and Bartlett’s test of sphericity was significant, χ² (276) = 2964.71, *p* < .001, confirming the suitability of factor analysis. The exploratory factor analysis revealed a six-factor structure, accounting for 52.41% of the total variance. Factor loadings ranged from  .32 to  .83, demonstrating acceptable item–factor associations.

Confirmatory factor analysis was performed to test the six-factor structure of the eBMRQ-TR, using diagonally weighted least squares (DWLS) estimation for ordinal Likert-type data. Model fit indices for the six-factor CFA model were indicated by χ² (237) = 628, *p* < .001, CFI = .946, TLI = .938, NFI = .917, RMSEA = .079 (90% CI = [.072,  .087]), and SRMR = .057. These results suggested that the six-factor structure showed an acceptable fit of the data. Inspection of modification indices exhibited no theoretically justified model changes; therefore, the model was used in its initial format.

**Fig 1:**
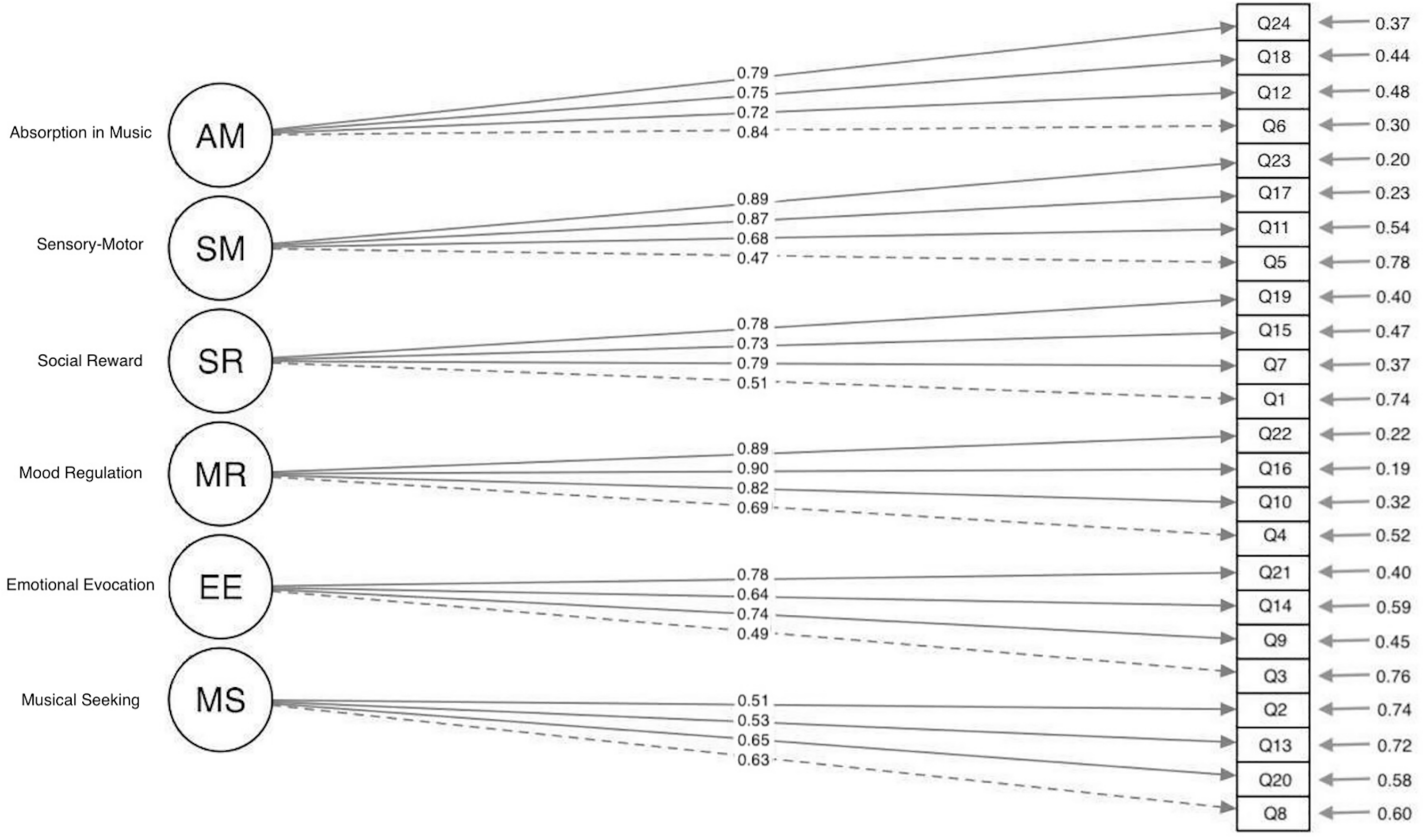
Confirmatory factor analysis model of the 24-item eBMRQ-TR.

The standardized path diagram of the six-factor CFA model is shown in Fig 1. Standardized factor loadings (λ) varied from  .47 to  .90, with all items exceeding the minimum acceptable loading criterion of  .40 [32,42]. Overall, the factor loadings demonstrated adequate to strong factor-item associations. Inter-factor correlations varied from  .73 to  .96, showing strong associations among the factors (see S1 Table for standardized factor loadings for the eBMRQ-TR and see S2 Table for inter-factor correlation matrix).

Convergent and discriminant validity were further investigated based on composite reliability (CR), average variance extracted (AVE), and the square root of AVE. CR values ranged from  .66 to  .90, while AVE values ranged between  .33 and  .69. Overall, CR values demonstrated acceptable internal consistency among the factors, while AVE values met or approached the recommended value of  .50 for most factors (see S3 Table for convergent and discriminant validity indices).

### Reliability analyses

Internal consistency was evaluated for both subscales and the total eBMRQ-TR score. The six subscales showed acceptable to good internal consistency, with Cronbach’s α ranging from  .60 to  .84 and McDonald’s ω from  .60 to  .85. The Musical Seeking subscale indicated relatively lower reliability, but within an acceptable range (α = .60, ω = .60). In contrast, good reliability was revealed for the other subscales. The internal consistency of the total eBMRQ-TR was excellent (α = .92; ω = .93). Test–retest reliability across a three-week interval was evaluated using intraclass correlation coefficients (ICCs) in a subsample of 65 participants. The ICCs showed moderate to good test-retest reliability across most subscales, while the Sensory-Motor subscale demonstrated lower temporal stability. Table 3 presents reliability coefficients and test-retest reliability for individual subscales and the total eBMRQ-TR score.

**Table 3:** Internal consistency and test-retest reliability of eBMRQ-TR.

Subscale	Internal consistency		Test-retest reliability	
	Cronbach’s α	McDonald’s ω	ICC	95% CI
Musical Seeking	.60	.60	.60	[.37, .71]
Emotional Evocation	.66	.68	.58	[.40, .72]
Mood Regulation	.84	.85	.80	[.68, .87]
Social Reward	.75	.75	.81	[.71, .88]
Sensory-Motor	.75	.76	.45	[.23, .62]
Absorption	.81	.81	.78	[.66, .86]
Total scale	.92	.93	.80	[.71, .86]

Note. α = Cronbach’s alpha*;* ω = McDonald’s omega*;* ICC = intraclass correlation coefficient for test-retest reliability over a three-week interval (n = 65); CI = confidence interval.

### Exploratory group comparisons

Findings from group comparisons across musicianship status for the eBMRQ-TR are reported in Table 4. Significant between-group differences were identified for five scales and the total eBMRQ score (*p* < .01); however, no significant differences were found for the Sensory-Motor scale (*p* = .22, η² = .01). Effect sizes varied between small to large (η² = .04 − .17). Post hoc comparisons revealed that both amateur and professional musicians reported significantly higher scores compared to non-musicians. For gender comparisons between groups, Welch’s *t* test showed a significant difference in total eBMRQ-TR scores between female and male participants, *t* (227.10) = 2.89, p = .003. Females had higher eBMRQ-TR scores (*M* = 3.82, *SD* = .62) than males (M = 3.57, SD = .78). Effect size ranged from small to moderate (Cohen’s d = .36). Additionally, a significant negative relationship was found between age and total eBMRQ-TR scores (Spearman’s *ρ* = −  .15, p = .015).

**Table 4:** Group comparisons across musicianship status of the eBMRQ-TR scores.

Subscale	Group	*n*	*M ± SD*	*F (p)*	η²	Post-hoc comparisons	Cohen’s *d*
MS	NM	189	3.01 ± .79	26.22 (<.001)	.17	NM < A*** NM < P***	0.84 1.25
	A	57	3.66 ± .72				
	P	20	4.01 ± .88				
EE	NM	189	3.83 ± .80	6.04 (.003)	.04	NM < A* NM < P**	0.35 0.66
	A	57	4.10 ± .67				
	P	20	4.35 ± .70				
MR	NM	189	4.07 ± .85	6.30 (.002)	.05	NM < A***	0.46
	A	57	4.44 ± .58				
	P	20	4.48 ± .72				
SR	NM	189	3.44 ± .89	14.60 (<.001)	.10	NM < A*** NM < P***	0.66 0.90
	A	57	4.02 ± .83				
	P	20	4.25 ± 1.03				
SM	NM	189	3.68 ± .95	1.5 (.22)	.01	–	–
	A	57	3.91 ± .78				
	P	20	3.84 ± .68				
AM	NM	189	3.31 ± .95	18.69 (<.001)	.13	NM < A*** NM < P***	0.66 1.13
	A	57	3.92 ± .85				
	P	20	4.36 ± .72				
Total scale	NM	189	3.50 ± .70	16.37 (<.001)	.11	NM < A*** NM < P***	0.67
	A	57	4.01 ± .54				
	P	20	4.21 ± .64				

Note. Scores are reported as mean ± standard deviations. Effect sizes are noted as eta squared (*η²*) and Cohen’s *d.* NM: Non-musician, A: Amateur musician, P: Professional musician. MS = Musical Seeking; EE = Emotional Evocation; MR = Mood Regulation; SR = Social Reward; SM = Sensory-Motor; AM = Absorption. Only significant post-hoc comparisons are indicated (* p <  .05, ** p <  .01, ***p <  .001).

## Discussion

The present study examined the validity and reliability of the Turkish version of the extended Barcelona Music Reward Questionnaire (eBMRQ-TR). The findings supported a six-factor model and demonstrated acceptable reliability and validity among Turkish-speaking adults. Furthermore, the scale provided additional evidence of the eBMRQ’s temporal stability over a three-week period.

The current findings for the six-factor eBMRQ-TR were consistent with those reported in previous language adaptations of both the BMRQ and the extended BMRQ. Most cross-cultural BMRQ validity studies, such as the French [23], Brazilian Portuguese [25], Danish [26], and Chinese [24] adaptations, have focused on the original five-factor BMRQ structure, whereas the extended BMRQ has been investigated primarily in the Italian population [27]. Accordingly, the present study used the extended version of the BMRQ, which added Absorption in Music as a sixth dimension to evaluate musical reward in a broader context. The Absorption dimension captures how deeply individuals engage with music, reflecting individual differences in sustained attention and immersion during music listening. Furthermore, all subscales used in the eBMRQ-TR together covered a comprehensive range of perceptual, emotional, and motivational dimensions of musical reward. Collectively, eBMRQ-TR reflected various aspects of musical experience, highlighting individual differences that can affect musical reward.

### Validity of the eBMRQ-TR

The construct validity of the eBMRQ-TR was confirmed through EFA and CFA. The EFA revealed a six-factor structure that aligns with the eBMRQ-TR, and the CFA supported construct validity by demonstrating that the proposed latent structure satisfactorily explained the item responses. Overall, these results proposed that the eBMRQ-TR evaluates multiple aspects of musical reward in Turkish-speaking adults. In this study, the CFA goodness-of-fit indices were within the acceptable range and yielded results that were broadly consistent with those reported in prior BMRQ adaptations across languages [24,25,46], particularly with the Italian adaptation, which used eBMRQ [27].

At the item level, all items were retained for the eBMRQ-TR, consistent with prior BMRQ adaptation studies that used the full set of items [23,25,27]. One item showed slightly lower corrected item-total correlation (*r* = .29); however, it was retained because its factor loading (λ = 0.47) was acceptable and supported the overall model fit, in line with the procedure for multidimensional instruments [35,42]. The current findings, therefore, supported the structural coherence and construct validity of the eBMRQ-TR. The factor loadings in this study were aligned with those reported in both the Italian version of the eBMRQ and the original BMRQ development research [13,27,28]. Specifically, factor loadings for the current study and Italian adaptation were similar; items showed generally moderate to strong loadings on their respective factors, confirming the stability of the six-factor structure across languages [27]. Although the CFA strategies varied across studies [13,27,28], the stable item-factor relationships indicated a consistent six-factor structure suitable for applied research settings. Notably, the sixth factor, Absorption in Music, showed consistently robust item loadings in both Italian and Turkish adaptations, highlighting its role as a key component of musical reward experiences.

Convergent and discriminant validity were evaluated using CFA-based indices, as no directly comparable Turkish measure of individual differences in musical reward was available. Regarding convergent validity, CR values demonstrated satisfactory internal consistency across subscales, and AVE values generally within acceptable ranges, suggesting adequate convergent validity. Although some subscales demonstrated AVE values below the recommended cutoff of  .50 [47], and a small number of items did not meet the minimum acceptable or good model thresholds, these results probably indicated the multidimensional nature of these constructs. Considering these conditions, acceptable composite reliability levels were considered sufficient to demonstrate convergent validity [32, 47]. Inter-factor correlations indicated that several subscales were closely linked, particularly those related to motivation, social engagement, and musical absorption. Although the correlations were relatively high, the overall pattern supported acceptable discriminant validity, proposing that six subscales represented multidimensional but highly interconnected aspects of musical reward.

### Reliability of the eBMRQ-TR

The overall eBMRQ-TR score showed excellent internal consistency (α = .92, ω = .93), confirming the scale’s reliability as a comprehensive measure of musical reward. This reliability pattern was consistent with multiple cross-cultural BMRQ studies, which generally demonstrated acceptable to strong internal consistency across subscales and the total BMRQ scores [23,25–27]. Recent BMRQ validation studies have shown good reliability, both within ordinal modeling (as indicated by ordinal α/ω) and factor-score reliability methods, e.g., ORION [26,27]. The current evidence, therefore, supports the construct’s strength across different languages and analytical approaches.

At the subscale level, Cronbach’s α ranged from  .60 to  .84, and McDonald’s ω from  .60 to  .85, showing acceptable to good internal consistency across six dimensions in this study. Similarly, prior BMRQ adaptation studies have generally reported acceptable-to-good internal consistency for subscales, with reliability coefficients of  .60 −  .80 across dimensions [23,25,27]. The current findings also showed that the Mood Regulation, Sensory-Motor, and Absorption in Music subscales exhibited good reliability; Musical Seeking and Emotional Evocation demonstrated lower, yet still acceptable reliability. Similarly, for the Musical Seeking dimension, the Brazilian Portuguese adaptation also showed lower reliability (α = .63), suggesting that music-seeking tendencies, which may reflect the construct’s complexity [25]. Overall, these reliability findings probably indicate the varied, experience-based characteristics of these constructs, encompassing subjective emotional reactions to music and various types of active sound engagement, which are naturally diverse and influenced by individual differences in music perception [48,49].

The test-retest reliability of the eBMRQ-TR was assessed after a three-week interval. The current findings showed moderate to good temporal stability across eBMRQ-TR subscales, with ICC values ranging from  .45 to  .81. Mood Regulation, Social Reward, and Absorption in Music dimensions exhibited good test-retest reliability; while Musical Seeking, Emotional Evocation, and Sensory-Motor demonstrated relatively lower yet still acceptable consistency over time. Relatively low ICC values on some subscales may reflect the situation- and context-dependent nature of specific aspects of musical engagement. Specifically, the Sensory-Motor subscale tends to fluctuate with situational and physiological factors, whereas Musical Seeking shows heterogeneous, music-seeking tendencies. Emotional Evocation can change depending on current emotional and attentional conditions [13]. Collectively, most subscales showed good test–retest reliability, whereas some subscales had moderate to low ICC scores (e.g., Sensory-Motor). This variability across subscales is consistent with the related literature, suggesting inherent construct features rather than measurement instability [1,13,50,51]. Importantly, the total eBMRQ-TR score demonstrated satisfactory test-retest reliability (ICC = .80), suggesting a consistent measure of musical reward that could be potentially beneficial in applied settings that include repeated assessments over time. To our knowledge, prior adaptation studies have mainly focused on internal consistency measures; however, test-retest reliability has not been investigated. The current study extends previous BMRQ research by demonstrating acceptable temporal stability using ICCs and provides additional evidence for the eBMRQ-TR’s reliability and potential use in clinical research contexts.

### Exploratory group comparisons

The exploratory analyses provided further insights into individual differences in musical reward. The results showed that musicians (professional or amateur) had higher total eBMRQ-TR scores than non-musicians, with a moderate group difference (*F* = 16.37, *p* < .001, η² = .11), supporting the idea that musical reward may be influenced by long-term auditory training and musical experiences [13,23,52]. The current study also indicated significant differences in musicianship between the groups across all subscales, except for the Sensory-Motor subscale (*p* = .22). These findings were consistent with previous BMRQ adaptation studies, which reported significant differences between musicians and non-musicians on Musical Seeking and Emotion Evocation, whereas no differences on the Sensory-Motor subscale [13,27]. As reported by Mas-Herrero et al. [13], higher scores on Musical Seeking and Emotional Evocation were associated with increased engagement in music-related activities and more intense emotional reactions to sound in musicians than in non-musicians. Specifically, the Sensory-Motor subscale assesses involuntary physical reactions to rhythmic sounds, including the urge to tap or move, and is considered to depend on automatic auditory-motor coupling mechanisms [53]. These mechanisms also exist in people without formal musical education and may be influenced by fundamental aspects of how we process rhythmic sounds rather than by acquired musical proficiency [50,53]. Maintaining sensorimotor entrainment could support the use of rhythm and movement-focused approaches in musical rehabilitation and auditory research involving music perception [48]. Collectively, the evidence suggests that musical experiences likely enhance pleasurable responses to music, thereby strengthening associations between neural regions involved in pleasure and reward processing [13,54].

Exploratory analyses further found that females had higher total eBMRQ-TR scores than males, consistent with previous BMRQ and eBMRQ research. These studies reported modest results and did not define reward constructs related to music [13,24,26,27]. In general, females tend to show stronger emotional responses to music than males, including greater increases in positive feelings and more robust blood pressure and heart rate changes [55,56]. Given prior findings, higher eBMRQ-TR scores in females likely reflect their increased sensitivity to musical reward compared to males. Finally, the total eBMRQ-TR scores revealed a weak negative correlation with age (*ρ* = −  .15, *p* = .015). These results aligned with previous BMRQ and eBMRQ studies, suggesting a slight decline in sensitivity to musical reward with aging [13,27,57], which may be linked to broader changes in music involvement or reward sensitivity across the lifespan [57].

### Strengths and limitations

Several key strengths of this study should be emphasized. Based on the literature, this study is the first Turkish-language adaptation of the extended version of the BMRQ. The present work provides a detailed psychometric assessment of the eBMRQ-TR, including the factor structure, construct validity, internal consistency, and test-retest reliability. Importantly, evaluating temporal stability using ICCs extends the prior BMRQ and eBMRQ literature, suggesting that eBMRQ-TR may be particularly informative in applied research contexts involving repeated measurements over time. Additionally, using the eBMRQ, which includes the Absorption in Music dimension, the current study enabled a more precise definition of musical reward experiences.

This study has some limitations that should be recognized. One limitation of this study is the evaluation of convergent and discriminant validity using CFA-based indices, as no validated Turkish instruments are available for closely related musical reward constructs. Another limitation of this study is the high inter-factor correlations, which may suggest a higher-order general music-reward factor and could be explored further through hierarchical modeling. A further limitation is the use of an online platform for data collection, which facilitates access to large samples [58]; however, this may lead to response bias due to limited interaction between researchers and participants [59]. This risk was mitigated by providing participants with a detailed information sheet that included clear instructions and a brief outline before they completed the scale. Using a community-based sample may limit the generalizability of the findings. For instance, the eBMRQ-TR has not yet been tested in clinical research involving individuals with hearing-related conditions. Future research may examine its suitability in these groups to better understand musical reward in both clinical and applied research contexts.

## Conclusions

The present study showed that the six-factor structure of the eBMRQ-TR is a valid and reliable questionnaire for measuring individual differences in musical reward among Turkish-speaking adults. The established ICC values of the eBMRQ-TR confirm its temporal stability and may be beneficial for repeated measurements in research settings. The higher eBMRQ-TR scores observed among musicians indicate that this scale is influenced by musical experience, which may make it suitable for use in clinical and research settings focused on music perception and reward. These findings may inform future research on the applicability of the eBMRQ-TR across populations, particularly in auditory research involving music perception and listening experiences.

## Supporting information

S1 File Turkish version of the eBMRQ. (DOCX)

S2 File Anonymized dataset of eBMRQ-TR responses. (XLSX)

S3 File Original English version of the eBMRQ. (DOC)

S1 Table Standardized factor loadings (λ) for the eBMRQ-TR. MS = Musical Seeking; EE = Emotion Evocation; MR = Mood Regulation; SR = Social Reward; SM = Sensory–Motor; AM = Absorption in Music.
(DOCX)

S2 Table Inter-factor correlation matrix across six subscales. Values represent standardized correlations among latent factors estimated via the CFA model; diagonal elements were fixed to 1.00. MS = Musical Seeking; EE = Emotional Evocation; MR = Mood Regulation; SR = Social Reward; SM = Sensory-Motor; AM = Absorption.
(DOCX)

S3 Table Convergent and discriminant validity indices (CR, AVE, and √AVE). λ: standardized factor loadings; CR = composite reliability; AVE = average variance extracted; √AVE = square root of AVE. √AVE values indicate discriminant validity.
(DOCX)
