# Supplementary material for: Reliability and validity of the Turkish version of the extended Barcelona Music Reward Questionnaire
Source: PLoS One. 2026 Jun 18;21(6):e0347517. doi: 10.1371/journal.pone.0347517 (PMC13278414; doi:10.1371/journal.pone.0347517)
Supplement: S1 File — (DOCX) [file pone.0347517.s001.docx]

**Genişletilmiş Barselona Müzik Hazzı Ölçeği**

Aşağıda yer alan ölçeğin her bir maddesi, kişinin katılacağı veya katılmayacağı bir ifadeyi belirtir. Her bir madde için, maddede söylenenlere ne kadar katıldığınızı veya katılmadığınızı belirtin. Lütfen tüm maddelere cevap verin, hiçbir maddeyi boş bırakmayın. Her bir maddeye yalnızca bir yanıt verin. Lütfen olabildiğince doğru ve içten yanıtlar verin. Her bir maddeye sanki tek bir maddeymiş gibi yanıt verin. Yani yanıtlarınızda tutarlı olma konusunda endişelenmeyin. Yanıtlarınızda kesinlikle katılmıyorum (sol) ile kesinlikle katılıyorum (sağ) arasında şu beş seçenekten birini seçin:

1: Kesinlikle katılmıyorum;

2: Katılmıyorum;

3: Ne katılıyorum ne de katılmıyorum (Kararsızım);

4: Katılıyorum;

5: Kesinlikle katılıyorum.

|  | 1 | 2 | 3 | 4 | 5 |
| --- | --- | --- | --- | --- | --- |
| 1. Birisiyle müzik paylaştığımda o kişiyle aramda özel bir bağ olduğunu hissederim. |  |  |  |  |  |
| 1. Boş zamanlarımda çok nadir müzik dinlerim. |  |  |  |  |  |
| 1. Duygu içeren müzikler dinlemeyi severim. |  |  |  |  |  |
| 1. Yalnızken müzik bana eşlik eder. |  |  |  |  |  |
| 1. Sevdiğim müzikle olsa dahi dans etmeyi sevmem. |  |  |  |  |  |
| 1. Bazen kendimi müzikle bütünleşmiş gibi hissederim. |  |  |  |  |  |
| 1. Müzik, diğer insanlarla bağ kurmamı sağlar. |  |  |  |  |  |
| 1. Sevdiğim müzikler hakkında bilgi edinip bir şeyler öğrenirim. |  |  |  |  |  |
| 1. Bazı müzik parçalarını dinlerken duygulanırım. |  |  |  |  |  |
| 1. Müzik beni sakinleştirir ve rahatlatır. |  |  |  |  |  |
| 1. Müzik beni çoğu zaman dans ettirir. |  |  |  |  |  |
| 1. Müzik dinlerken kendimi ve çevremi unutacak kadar dalıp giderim. |  |  |  |  |  |
| 1. Her zaman yeni müzikler ararım. |  |  |  |  |  |
| 1. Çok sevdiğim bir melodiyi dinlediğimde gözlerim dolabilir veya ağlayabilirim. |  |  |  |  |  |
| 1. Başkalarıyla birlikte şarkı söylemekten veya enstrüman çalmaktan hoşlanırım. |  |  |  |  |  |
| 1. Müzik sakinleşmeme yardımcı olur. |  |  |  |  |  |
| 1. Sevdiğim müziği mırıldanmaktan veya ona eşlik etmekten kendimi alamam. |  |  |  |  |  |
| 1. Bazen tamamen müziğe dalıp, bilincimin geçici olarak değiştiğini hissederim. |  |  |  |  |  |
| 1. Konserde kendimi sanatçılarla ve seyirciyle bağ kurmuş gibi hissederim. |  |  |  |  |  |
| 1. Müzik ve ilgili ürünlere oldukça fazla para harcarım. |  |  |  |  |  |
| 1. Bazen hoşuma giden bir melodiyi duyduğumda ürperecek kadar heyecanlanırım. |  |  |  |  |  |
| 1. Müzik beni rahatlatır. |  |  |  |  |  |
| 1. Çok hoşuma giden bir melodi duyduğumda, ona uygun ritim tutmaktan veya hareket etmekten kendimi alamam. |  |  |  |  |  |
| 1. Çok sevdiğim müzikler dinlerken bazen bulutların üstündeymiş gibi hissederim. |  |  |  |  |  |
