# Supplementary material for: Reliability and validity of the Turkish version of the extended Barcelona Music Reward Questionnaire
Source: PLoS One. 2026 Jun 18;21(6):e0347517. doi: 10.1371/journal.pone.0347517 (PMC13278414; doi:10.1371/journal.pone.0347517)
Supplement: S3 File — (DOC) [file pone.0347517.s003.doc]

**BMRQ**

Each item of this questionnaire is a statement that a person may either agree with or disagree with. For each item, indicate how much you agree or disagree with what the item says. Please respond to all the items; do not leave any blank. Choose only one response to each statement. Please be as accurate and honest as you can be. Respond to each item as if it were the only item. That is, do not worry about being consistent in your responses. Choose from completely disagree (left) to completely agree (right) one of the five options:

1: completely disagree;

2: disagree;

3: neither agree nor disagree;

4: agree;

5: completely agree.

|  |  |  |  | 1 |  | 2 |  | 3 |  | 4 |  | 5 |  |
| --- | --- | --- | --- | --- | --- | --- | --- | --- | --- | --- | --- | --- | --- |
| 1 |  | When I share music with someone I feel a special connection with that person. |  |  |  |  |  |  |  |  |  |  |  |
| 2 |  | In my free time I hardly listen to music. |  |  |  |  |  |  |  |  |  |  |  |
| 3 |  | I like listen to music that contains emotion. |  |  |  |  |  |  |  |  |  |  |  |
| 4 |  | Music keeps me company when I’m alone. |  |  |  |  |  |  |  |  |  |  |  |
| 5 |  | I don’t like to dance, not even with music I like. |  |  |  |  |  |  |  |  |  |  |  |
| 6 |  | I sometimes feel like I am ‘one’ with the music. |  |  |  |  |  |  |  |  |  |  |  |
| 7 |  | Music makes me bond with other people. |  |  |  |  |  |  |  |  |  |  |  |
| 8 |  | I inform myself about music I like. |  |  |  |  |  |  |  |  |  |  |  |
| 9 |  | I get emotional listening to certain pieces of music. |  |  |  |  |  |  |  |  |  |  |  |
| 10 |  | Music calms and relaxes me. |  |  |  |  |  |  |  |  |  |  |  |
| 11 |  | Music often makes me dance. |  |  |  |  |  |  |  |  |  |  |  |
| 12 |  | While listening to music, I may become so involved that I may forget about myself and my surroundings. |  |  |  |  |  |  |  |  |  |  |  |
| 13 |  | I’m always looking for new music. |  |  |  |  |  |  |  |  |  |  |  |
| 14 |  | I can become tearful or cry when I listen to a melody that I like very much. |  |  |  |  |  |  |  |  |  |  |  |
| 15 |  | I like to sing or play an instrument with other people. |  |  |  |  |  |  |  |  |  |  |  |
| 16 |  | Music helps me chill out. |  |  |  |  |  |  |  |  |  |  |  |
| 17 |  | I can’t help humming or singing along to music that I like. |  |  |  |  |  |  |  |  |  |  |  |
| 18 |  | It is sometimes possible for me to be completely immersed in music and to feel as if my whole state of consciousness has been temporarily altered. |  |  |  |  |  |  |  |  |  |  |  |
| 19 |  | At a concert I feel connected to the performers and the audience. |  |  |  |  |  |  |  |  |  |  |  |
| 20 |  | I spend quite a bit of money on music and related items. |  |  |  |  |  |  |  |  |  |  |  |
| 21 |  | I sometimes feel chills when I hear a melody that I like. |  |  |  |  |  |  |  |  |  |  |  |
| 22 |  | Music comforts me. |  |  |  |  |  |  |  |  |  |  |  |
| 23 |  | When I hear a tune I like a lot I can’t help tapping or moving to its beat. |  |  |  |  |  |  |  |  |  |  |  |
| 24 |  | When listening to great music I sometimes feel as if I am being lifted into the air. |  |  |  |  |  |  |  |  |  |  |  |
