# Supplementary material for: Reliability and validity of the Turkish version of the extended Barcelona Music Reward Questionnaire
Source: PLoS One. 2026 Jun 18;21(6):e0347517. doi: 10.1371/journal.pone.0347517 (PMC13278414; doi:10.1371/journal.pone.0347517)
Supplement: S1 Table — MS = Musical Seeking; EE = Emotion Evocation; MR = Mood Regulation; SR = Social Reward; SM = Sensory–Motor; AM = Absorption in Music. (DOCX) [file pone.0347517.s004.docx]

**S1 Table. Standardized factor loadings (λ) for the eBMRQ-TR.**

| **Items** | **SR** | **MS** | **EE** | **MR** | **SM** | **AM** |
| --- | --- | --- | --- | --- | --- | --- |
| eBMRQ_2 |  | .51 |  |  |  |  |
| eBMRQ_13 |  | .53 |  |  |  |  |
| eBMRQ_8 |  | .63 |  |  |  |  |
| eBMRQ_20 |  | .65 |  |  |  |  |
| eBMRQ_3 |  |  | .49 |  |  |  |
| eBMRQ_9 |  |  | .74 |  |  |  |
| eBMRQ_14 |  |  | .64 |  |  |  |
| eBMRQ_21 |  |  | .78 |  |  |  |
| eBMRQ_4 |  |  |  | .69 |  |  |
| eBMRQ_10 |  |  |  | .82 |  |  |
| eBMRQ_16 |  |  |  | .90 |  |  |
| eBMRQ_22 |  |  |  | .89 |  |  |
| eBMRQ_1 | .51 |  |  |  |  |  |
| eBMRQ_7 | .79 |  |  |  |  |  |
| eBMRQ_15 | .73 |  |  |  |  |  |
| eBMRQ_19 | .78 |  |  |  |  |  |
| eBMRQ_5 |  |  |  |  | .47 |  |
| eBMRQ_11 |  |  |  |  | .68 |  |
| eBMRQ_17 |  |  |  |  | .87 |  |
| eBMRQ_23 |  |  |  |  | .89 |  |
| eBMRQ_6 |  |  |  |  |  | .84 |
| eBMRQ_12 |  |  |  |  |  | .72 |
| eBMRQ_18 |  |  |  |  |  | .75 |
| eBMRQ_24 |  |  |  |  |  | .79 |

Note*.* MS = Musical Seeking; EE = Emotion Evocation; MR = Mood Regulation; SR = Social Reward; SM = Sensory–Motor; AM = Absorption in Music.
