# Supplementary material for: Reliability and validity of the Turkish version of the extended Barcelona Music Reward Questionnaire
Source: PLoS One. 2026 Jun 18;21(6):e0347517. doi: 10.1371/journal.pone.0347517 (PMC13278414; doi:10.1371/journal.pone.0347517)
Supplement: S2 Table — Values represent standardized correlations among latent factors estimated via the CFA model; diagonal elements were fixed to 1.00. MS = Musical Seeking; EE = Emotional Evocation; MR = Mood Regulation; SR = Social Reward; SM = Sensory-Motor; AM = Absorption. (DOCX) [file pone.0347517.s005.docx]

**S2 Table: Inter-factor correlation matrix across six subscales.**

| **Subscale** | **MS** | **EE** | **MR** | **SR** | **SM** | **AM** |
| --- | --- | --- | --- | --- | --- | --- |
| MS | **1.00** |  |  |  |  |  |
| EE | .75 | **1.00** |  |  |  |  |
| MR | .77 | .78 | **1.00** |  |  |  |
| SR | .92 | .77 | .77 | **1.00** |  |  |
| SM | .70 | .73 | .82 | .73 | **1.00** |  |
| AM | .96 | .90 | .82 | .89 | .81 | **1.00** |

**Note*.*** Values represent standardized correlations among latent factors estimated via the CFA model; diagonal elements were fixed to 1.00. MS = Musical Seeking; EE = Emotional Evocation; MR = Mood Regulation; SR = Social Reward; SM = Sensory-Motor; AM = Absorption*.*
