# Supplementary material for: Reliability and validity of the Turkish version of the extended Barcelona Music Reward Questionnaire
Source: PLoS One. 2026 Jun 18;21(6):e0347517. doi: 10.1371/journal.pone.0347517 (PMC13278414; doi:10.1371/journal.pone.0347517)
Supplement: S3 Table — λ: standardized factor loadings; CR = composite reliability; AVE = average variance extracted; √AVE = square root of AVE. √AVE values indicate discriminant validity. (DOCX) [file pone.0347517.s006.docx]

**S3 Table: Convergent and discriminant validity indices (CR, AVE, and √AVE).**

| **Factor** | **λ**  **(range)** | **CR** | **AVE** | **√AVE** |
| --- | --- | --- | --- | --- |
| MS | .51–.65 | .66 | .33 | .57 |
| EE | .49–.78 | .74 | .44 | .66 |
| MR | .69–.90 | .90 | .69 | .83 |
| SR | .51–.79 | .78 | .48 | .69 |
| SM | .47–.89 | .81 | .53 | .73 |
| AM | .72–.84 | .85 | .60 | .78 |

**Note.** λ: standardized factor loadings; CR = composite reliability; AVE = average variance extracted; √AVE = square root of AVE. √AVE values indicate discriminant validity.
